# Prevention of Loperamide-Induced Constipation in Mice and Alteration of 5-Hydroxytryotamine Signaling by *Ligilactobacillus salivarius* Li01

**DOI:** 10.3390/nu14194083

**Published:** 2022-10-01

**Authors:** Bo Qiu, Lian Zhu, Shuobo Zhang, Shengyi Han, Yiqiu Fei, Furong Ba, Björn Berglund, Lanjuan Li, Mingfei Yao

**Affiliations:** 1 State Key Laboratory for Diagnosis and Treatment of Infectious Diseases, National Clinical Research Center for Infectious Diseases, Collaborative Innovation Center for Diagnosis and Treatment of Infectious Diseases, The First Affiliated Hospital, School of Medicine, Zhejiang University, Hangzhou 310003, China; 2 School of Basic Medical Sciences and Forensic Medicine, Hangzhou Medical College, Hangzhou 310053, China; 3 Department of Biomedical and Clinical Sciences, Linköping University, SE-58183 Linköping, Sweden; 4 Jinan Microecological Biomedicine Shandong Laboratory, Jinan 250021, China

**Keywords:** constipation, *Ligilactobacillus salivarius* Li01, 5-hydroxytryotamine, gut microbiota

## Abstract

Although *Ligilactobacillus salivarius* Li01 (Li01) has shown much promise in preventing multiple gastrointestinal diseases, the potential of the probiotic in alleviating constipation and the related mechanisms remain unclear. In this study, the effects of Li01 were evaluated in a loperamide-induced constipation mouse model. The results demonstrated that Li01 intervention can relieve constipation symptoms by improving water content, quantity, and morphology of feces and act as an intestinal barrier structure protector. Furthermore, Li01 can modulate gut motility (gastrointestinal transit rate), the fluid transit-associated expression of aquaporins, and the serum parameters vasoactive intestinal peptide, substance P, and somatostatin. Constipation significantly increased the levels of 5-hydroxytryotamine (5-HT) in serum (*p* < 0.01) and decreased the levels in the intestine (*p* < 0.001). Due to its function of elevating the expression of tryptophan hydroxylase 1, this was reversed after Li01 treatment. Li01 also promoted the expression of 5-HT receptor 3 and 4, indicating that the 5-HT signaling pathway may play a critical role in the mechanism by which Li01 alleviate constipation symptoms. Additionally, Li01 significantly altered the gut microbiota composition by enhancing the ratio of *Firmicutes*/*Bacteroidetes* and increasing the abundance of *Rikenellaceae_RC9* genera. Based on the above results, Li01 may have the potential to effectively alleviate constipation by regulating the 5-HT pathway and alteration of the gut microbiota.

## 1. Introduction

Constipation is one of the most common functional gastrointestinal (GI) disorders and is characterized by persistently difficult and infrequent defecation, often accompanied by uncomfortable symptoms including dry stools, excessive straining, abdominal pain, and bloating [1]. Currently, the global prevalence of persons suffering from constipation, which seriously influences their life quality and mental status, is around 15% and increasing [2]. Various factors are associated with the occurrence of constipation, such as adverse drug reactions (e.g., opioid pain medication), systemic illness (e.g., Parkinson’s disease), local pathology (e.g., colorectal cancer), intestinal nervous system dysfunction, lifestyles, and eating patterns [3]. It is believed that gut motility, fluid transportation, and alteration of the microbiota are the main mechanisms by which constipation occurs [4].

There is a close relationship between gut microbiota and constipation; recent studies have indicated that there are significant differences in gut microbiota composition between healthy individuals and patients with functional constipation [5]. Compared with germ-free mice, mice colonized with gut microbiota have shown higher colonic contractility and significantly lower GI transit time [6]. The gut microbiota can regulate the level of serotonin (5-hydroxytryptamine, 5-HT) by improving the expression of tryptophan hydroxylase 1 (Tph1) in the host intestinal tract [7]. 5-HT is mainly synthesized in enterochromaffin (EC) cells and secreted by mucosal mast cells and myenteric neurons [8]. As one of the gut neurotransmitters, 5-HT can mediate GI functions through activating multiple 5-HT receptors. Among them, 5-HT receptor 3 (5-HTR3) and 4 (5-HTR4) play a vital role in peristaltic reflexes and intestinal propulsion [9,10]. Moreover, 5-HTR4 also influences colonic anion and fluid secretion, which may contribute to the relief of constipation [11]. Additionally, gut microbes can indirectly affect constipation through the production of metabolites such as short-chain fatty acids and peptides, which stimulate the enteric nervous system and promote GI transit [12].

Probiotics have been shown to have the potential in use as a treatment for constipation. Probiotics are effective laxatives that can ameliorate constipation with fewer side effects compared to chemical drugs [13]. In one double-blinded, placebo-controlled, randomized trial, it was shown that multi-strain probiotics considerably promoted spontaneous bowel movements in constipated patients [14]. Recently, the *Bacillus coagulans* strain SNZ 1969 has been confirmed to contribute to ameliorating constipation by improving gut motility. Researchers have also found that decreased colonic transit time is related to the enriched abundance of *Lactobacillales* and the diminishment of the *Synergistales* genera in the gut microbiota [15]. Our previous research has also indicated that oral administration of *Ligilactobacillus salivarius* Li01 (Li01) facilitated intestinal barrier recovery and gut microbiota restoration [16]. In addition, Li01 has also been reported to be able to decrease the serum level of inflammatory cytokines and prevent bacterial translocations [17]. Our recent study found that Li01 was related to the regulation of the 5-HT signaling pathway and promoted 5-HT synthesis [18]. Since several studies have shown the potential of Li01 in preventing GI diseases, it can be inferred and suspected that the probiotic could be a promising agent for constipation therapy.

In this study, the effect of Li01 on constipation was investigated. Loperamide hydrochloride was used to establish a constipation mouse model. A probiotic, solid beverage product, mainly composed of *Lactobacillus helveticus* and *Bifidobacterium longum* was used as positive control. The fecal parameters, gut motility, fluid transit, serum indicators, histopathology, and the compositional change of the microbiota in feces were analyzed. Moreover, we investigated the alteration of the 5-HT signaling pathway in order to clarify the mechanism by which Li01 affects GI function. This study evaluated the therapeutical, preventative effect of Li01 on constipation in mice and illustrated the related mechanism of action.

## 2. Materials and Methods

### 2.1. Bacterial Cultivation

Li01 was cultured in De Man, Rogosa, and Sharpe (MRS) broth (Oxoid, Basingstoke, Hampshire, UK) at 37 °C for 24 h in an anaerobic chamber (Electrotek Scientific, West Yorkshire, UK). Before oral gavage, the bacterial cells were collected by centrifugation at 4000 rpm for 5 min at 4 °C. After centrifugation, the supernatant was removed, and the bacterial pellet was washed with saline buffer twice. The bacterial cells were then resuspended in saline buffer to obtain a concentration of ~10^10^ CFU/mL. Weileshu (WLS), a probiotic solid beverage product composed of *Lactobacillus helveticus* and *Bifidobacterium longum* (kindly provided by Shaoxing Tongchuang Biological Technology Co., Ltd., Shaoxing, China) was dissolved in saline buffer before use according to the recommended dose. Both of these strains have been reported to have the effect of relieving constipation [19,20].

### 2.2. Establishment of Animal Models and Experimental Design

**Figure 1:**
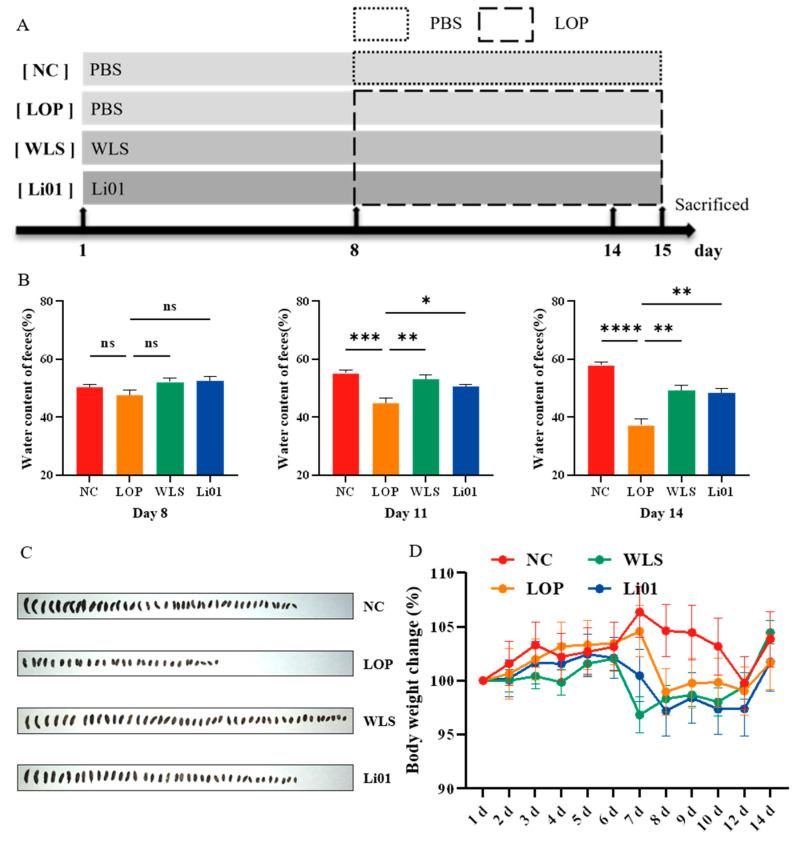
Changes in fecal characteristics and body weight in mice with loperamide-induced constipation (LOP) after treatment with *Ligilactobacillus salivarius* Li01 (Li01), Weileshu probiotic (WLS), and the control group (NC). (**A**) Flow chart of the animal experiments. (**B**) Water content of feces on the 8th, 11th, and 14th day. (**C**) Fecal morphology after induction of constipation. (**D**) Body weight changes of mice in the different experimental groups. Data are presented as mean ± SEM, *n* = 6. Significant differences are denoted by “ns” (*p* ≥ 0.05), * (*p* < 0.05), ** (*p* < 0.01), *** (*p* < 0.001), and **** (*p* < 0.0001).

As shown in Figure 1A, 24 C57BL/6 male mice (specific-pathogen-free, 5-week-old) were obtained from Shanghai BK Company (Shanghai, China). All mice were fed with a standard AIN93G diet with accessible food and water ad libitum. After acclimation for one week, mice were randomly divided into four groups (*n* = 6). The groups were the normal control group (NC), the loperamide-induced constipation group (LOP), the constipated mice with the WLS treatment group (WLS), and the constipated mice with the Li01 treatment group (Li01). The experiment was divided into two stages: the probiotic pre-treatment stage and the loperamide-induced constipation stage. In the first stage, mice in the NC group and the LOP group received a daily gavage of 0.4 mL saline buffer for 15 days, whereas mice in the Li01 group and the WLS group received a daily gavage of 0.4 mL Li01 solution (~10^10^ CFU/mL) and 0.4 mL WLS solution (0.5 g/mL) for 15 days, respectively. On the 8th–15th day, mice in the NC group also received a daily gavage of 0.2 mL saline buffer, and mice in the remaining groups received a daily gavage of 0.2 mL loperamide hydrochloride (10 mg/kg body weight) (Sigma-Aldrich, St. Louis, MO, USA) instead of saline, to induce constipation from the 8th to 15th day. The weight of all mice was recorded daily during the experiment. On the 15th day, all mice were sacrificed, and the samples of blood, small intestine, colonic tissue, and intestinal contents were collected for subsequent analysis.

### 2.3. Measurement of Water Content in Mouse Feces

On the 8th, 11th, and 14th days, after the daily gavage, mice in different groups were transferred to separate, clean cages lined with absorbent papers. After 2 h, the feces in the cages were collected, and wet weight of the feces was immediately measured. Then, the same collected feces were dried at 70 °C for 18 h, and the dry weight of the feces was recorded. The water content of the feces was calculated according to the following formula:

Water content of the feces (%) = (wet weight-dry weight)/wet weight × 100%

### 2.4. Determination of GI Transit Rate

To analyze the GI transit rates, another 24 C57BL/6 male mice were purchased (Shanghai BK Company) and randomly divided into four groups (*n* = 6): NC, LOP, WLS, and Li01 groups. On the 1st–14th day, the experiment design was the same as described above. An activated carbon solution was prepared in accordance with the previously reported method and stored at 4 °C for use [21]. On the 14th day, all mice were fasted overnight but were not deprived of water. On the 15th day, each mouse received oral gavage of an 0.2 mL activated carbon solution. After 20 min, all mice were sacrificed, and the GI tract tissues were collected. The entire length of the small intestine and the migration distance traveled by activated carbon from the pylorus to the front end of the activated carbon were determined. The GI transit rate was calculated according to the following formula:

GI transit rate (%) = migration distance of activated carbon/entire length of the small intestine × 100%

### 2.5. Biochemical Analysis

The collected blood samples were placed for 2 h at room temperature before centrifugation at 3000× *g* for 15 min at 4 °C to obtain serum samples. Tissue samples were collected by weighing approximately 100 mg of colonic tissue from the mice, mixing with pre-cooled phosphate-buffered saline at the ratio of 1:9, followed by homogenization and centrifugation at 10,000× *g* for 10 min at 4 °C. The concentration of proteins in the tissue homogenate was quantified using a BCA protein assay kit (Beyotime, Shanghai, China). All serum and tissue homogenate samples were separated and stored at −80 °C. The serum level of vasoactive intestinal peptide (VIP), substance P (SP), gastrin (GAS), somatostatin (SST), and 5-HT, and the tissue level of 5-HT were determined by using ELISA kits (Elabscience Biotechnology Co. Ltd., Wuhan, China).

### 2.6. Quantitative PCR Assay

The total RNA of the colonic tissue was extracted using TRIzol reagent (Invitrogen, Carlsbad, CA, USA), and subsequently converted to cDNA with the PrimeScript RT Master MiX (TaKaRa, Kusatsu, Shiga, Japan). Then the cDNA samples from each group were deleted by TB Green Premix Ex Taq II kit (TaKaRa, Kusatsu, Shiga, Japan), and the reaction was performed in QuantStudio 5 Quantitative Real-Time PCR System (Applied Biosystems, Foster City, CA, USA). The sequences of amplified primers (GAPDH, AQP3, AQP4, Muc2, Tph1, SERT, 5-HTR3, and 5-HTR4) are displayed in Table 1. The changes in gene expression were analyzed by the 2−ΔΔCT method.

### 2.7. Histological Analysis

Segments of distal colonic tissue (1 cm) collected from the mice were clipped and fixed with 10% neutral-buffered formalin (Hepeng Biology, Shanghai, China) for 24 h. Colonic tissue samples were embedded in paraffin and cut into cross sections with a thickness of 4 μm. The cross sections were then stained with hematoxylin and eosin for histological analysis. To prepare the samples for immunofluorescence analysis, the colonic sections were deparaffinized, soaked with 0.01 M Tris/EDTA (Sigma-Aldrich, St. Louis, MO, USA) for 10 min, and permeabilized in 0.1% Triton X-100 (Sigma-Aldrich, St. Louis, MO, USA). After the sections were blocked by PBS containing 5% BSA, they were incubated with the monoclonal primary antibody for 5-HT (1:500, GeneTex, Irvine, CA, USA). Finally, the sections were treated with the FITC-conjugated goat anti-mouse secondary antibody (Beyotime, Shanghai, China) and DAPI (Life Technologies, Carlsbad, CA, USA). Immunofluorescent images were acquired by using laser scanning confocal microscopy (Leica, Wetzlar, Germany). Five fields were randomly selected from each section, and the thickness of muscle and the depth of crypts were measured using Caseviewer software [22].

### 2.8. 16S rRNA Gene Analysis

Intestinal content samples from the 15th day were collected in sterile microtubes and frozen at −80 °C. The E.Z.N.A. ^®^Stool DNA kit (D4015, Omega Bio-Tek, Inc., Norcross, GA, USA) was used to extract the genomic DNA of bacteria. The V3-V4 region of the bacterial small-subunit 16S rRNA was amplified with primers 341F (5′-CCTACGGGNGGCWGCAG-3′) and 805R (5′-GACTACHVGGGTATCTAATCC-3′). The PCR products were purified by AMPure XT beads (Beckman Coulter Genomics, Danvers, MA, USA) and quantified by Qubit (Invitrogen, Carlsbad, CA, USA). Purified amplicons were pooled in equimolar and paired-end sequenced (2 × 300) on an Illumina MiSeq platform. The trimmomatic software was used to generate paired-end reads. Then, operational taxonomic units (OTUs) were clustered with Vsearch software, and the representative read was selected through the QIIME2 package. PCoA analysis was conducted with R software.

### 2.9. Data Analysis

The data are presented as mean ± standard error of mean (SEM). Statistical analyses were performed by using GraphPad Prism 9.0 (GraphPad Software Inc., San Diego, CA, USA). One-way ANOVA (for data that passed the normality test) or the Kruskal–Wallis test (for data that failed the normality test) were used to test significant differences between groups. Figures were constructed by using GraphPad Prism 9.0 and R software. *p*-value < 0.05 was considered to be statistically significant.

## 3. Results and Discussion

### 3.1. Establishment of a Constipation Mouse Model and Effects of Treatment

Constipation has numerous symptoms, including hard or small stools, difficult defecation, and infrequent bowel movements [23]. Loperamide is an agonist of µ-opioid receptors that inhibits gut peristalsis, decreases the propulsion of the intestinal contents, and increases the absorption of water and ions [24]. The main side effects of loperamide are bloating and constipation, and its cardiovascular toxicity has also been reported [25]. In this study, loperamide was used to establish a constipation mouse model as in the previous studies [26,27,28] with some modifications. The fecal water content and fecal morphology (Figure 1B,C) are associated parameters by which to evaluate the function of the GI tract. On the eighth day, after oral gavage with loperamide for the first time, the water content of feces in all groups remained relatively stable. However, on the 11th day, the water content of feces in the LOP group showed a significant decrease compared with the NC group (*p* < 0.001) (Figure 1B). The reduced water content of feces increased the difficulty of defecation, and these mice also exhibited typical symptoms of constipation, consistent with results from a previous study [29]. Meanwhile, fecal water content was observed to increase in the constipated mice both after oral administration of Li01 (*p* < 0.05) and WLS (*p* < 0.01). The fecal quantity excreted in 2 h from the 11th day and their morphology were remarkably changed after constipation was induced (Figure 1C). Compared to mice in the NC group, the fecal quantity was considerably decreased, and the consistency of the feces were small, short, hard, and dry in the LOP group. However, treatment with Li01 and WLS reversed the fecal properties compared to the LOP group, in which increased fecal quantity was observed, and the fecal consistency was larger in size, softer, and wetter. On the 14th day, the water content of feces significantly decreased in the LOP group compared to the NC group (*p* < 0.0001), whereas no significant differences were found among the control, Li01, or WLS group. The results suggested that a constipation mouse model was successfully established and that the probiotic Li01 was able to relieve constipation-associated symptoms such as improving the water content of feces, fecal quantity, and morphology. However, no significant variation in body weight among different groups was found (Figure 1D).

### 3.2. Effects of Li01 on Gut Motility and Fluid Transit in Constipated Mice and Associated Parameters

**Figure 2:**
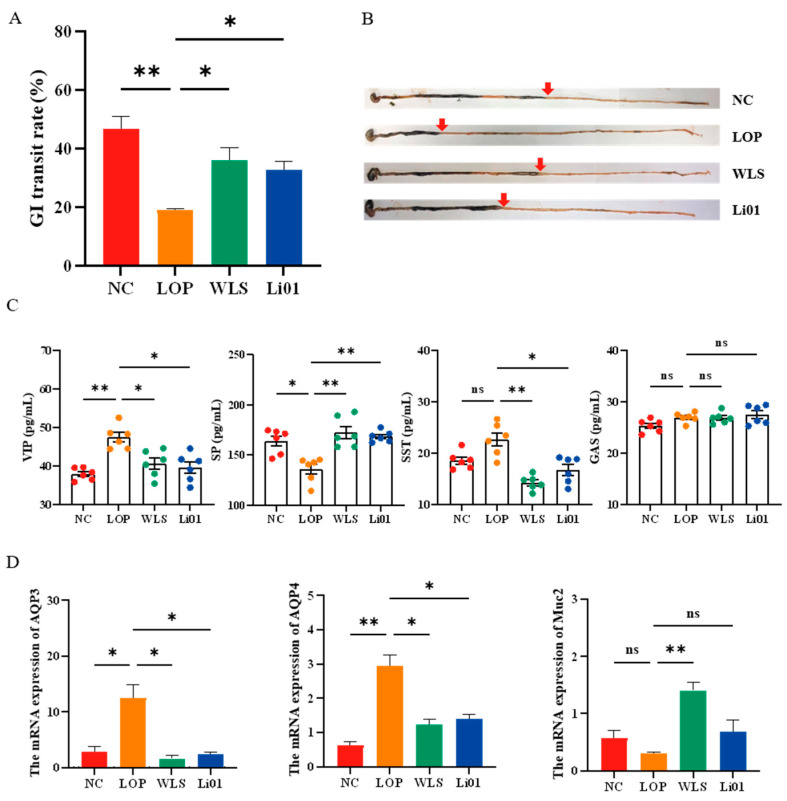
Changes in gut motility and fluid transit and the associated parameters in mice with loperamide-induced constipation (LOP) after treatment with *Ligilactobacillus salivarius* Li01 (Li01), Weileshu probiotic (WLS), and the control group (NC). (**A**) Gastrointestinal transit rate in mice in the NC, LOP, WLS, and Li01 groups after induction of constipation. (**B**) Recording of activated carbon GI transport in mice in the experimental groups. The red arrows indicate the front end of the activated carbon. (**C**) The serum levels of vasoactive intestinal peptide (VIP), substance P (SP), somatostatin (SST), and gastrin (GAS). (**D**) mRNA expression levels of intestinal aquaporin-3 (AQP3), aquaporin-4 (AQP4), and Mucin-2 (Muc2). Data are presented as mean ± SEM, *n* = 6. Significant differences are denoted by “ns” (*p* ≥ 0.05), * (*p* < 0.05), and ** (*p* < 0.01).

Previous research has found that one of the main causes leading to hard and dry stool is reduced gut motility, which may promote the accumulation of intestinal contents so that the intestinal absorption of water and electrolytes increases [30]. GI peristalsis includes coordinated contraction and relaxation of the intestinal muscle, participating in the formation of most gut propulsion motility [3]. GI transit rate can be used to evaluate the GI peristalsis in mice. According to the results shown in Figure 2A,B, the GI transit rate dramatically decreased in the LOP group (*p* < 0.01) compared with the NC group. Interestingly, compared to the LOP group, the GI transit rate was significantly higher in the Li01 (*p* < 0.05) and WLS groups (*p* < 0.05), indicating that oral administration of both Li01 and WLS increased the gut motility in constipated mice.

Gut motility and fluid transit are usually associated with imbalance of GI regulatory-related peptides, which participate in the regulation of intestinal function [4]. SP and GAS are the main excitatory transmitters in the enteric nervous system. SP can stimulate interstitial cells of Cajal and produce intense, intestinal smooth muscle contraction [31]. GAS is responsible for promoting the gastric acid release and for increasing antral muscle contractions and gut motility [32]. VIP and SST are considered to be inhibitory transmitters. VIP is a crucial regulator which can maintain the relaxation of the intestine and upregulate the secretion of intestinal fluid [33]. SST can aggravate constipation by inhibiting the release of GI hormones and slowing intestinal transit time [34]. The levels of SP, GAS, VIP, and SST in the serum of the mice in the experiment are shown in Figure 2C. Loperamide-induced constipation significantly increased the serum level of VIP (*p* < 0.01) and reduced the level of SP (*p* < 0.05). The serum level of SST was elevated compared with the NC group, but not to a statistically significant degree (*p* = 0.11). Constipated mice that had an Li01 intervention showed significantly decreased serum levels of VIP (*p* < 0.05) and SST (*p* < 0.01), and improved level of SP (*p* < 0.01). These effects of Li01 were similar among the positive control and the NC group. Additionally, no significant difference in serum levels of GAS was observed among any group. These results indicate that Li01 may increase gut motility and improve intestinal fluid secretion in constipated mice through modulation of the humoral regulation system, specifically adjusting the serum levels of gastrointestinal regulatory-related peptides such as VIP, SP, and SST.

Aquaporins, a family of water channel proteins, are closely associated with GI transit rate due to their crucial role in the osmotic modulation of water fluid homeostasis [35]. Since VIP can increase the expression of AQP3 by a cAMP-dependent pathway [36], and the results presented above showed increased levels of VIP during constipation, we speculate that the expressions of AQP3 and AQP4 were altered in constipated mice. AQP3 is mainly expressed in colonic tissue and is considered to affect water transport in the GI tract. Excessive expression of AQP3 can cause severe constipation [37]. AQP4 is normally located in the colonic epithelial cells. It has been shown that AQP4 is rehydration of intestinal contents [38]. As shown in Figure 2D, compared to the NC group, the LOP group had significantly higher levels of AQP3 (*p* < 0.05) and AQP4 (*p* < 0.01), whereas treatments of Li01 and WLS decreased levels of AQP3 (*p* < 0.05, *p* < 0.05) and AQP4 (*p* < 0.05, *p* < 0.05) compared to constipated mice, indicating that treatment with Li01 down-regulated the expression of AQP3 and AQP4, which can improve the water content of feces.

Mucin consists of glycoproteins with high molecular weight and contributes to the formation of the mucosal barrier in the intestinal epithelium and facilitate lubrication of the intestinal tract by preventing the loss of water [39]. Mucin 2 (Muc2) is the predominant secreted mucin from goblet cells. Although no significant difference in the expression of Muc2 could be observed between the NC, LOP, and Li01 groups, the levels of Muc2 were highly elevated in the WLS group compared to the LOP group (*p* < 0.05) (Figure 2D). These results demonstrate that intervention with WLS may have the potential to facilitate lubrication of the intestinal tract by increasing the expression of Muc2, and Li01 may not have this effect.

### 3.3. Effects of Li01 on the Intestinal Barrier Structure in Constipated Mice

**Figure 3:**
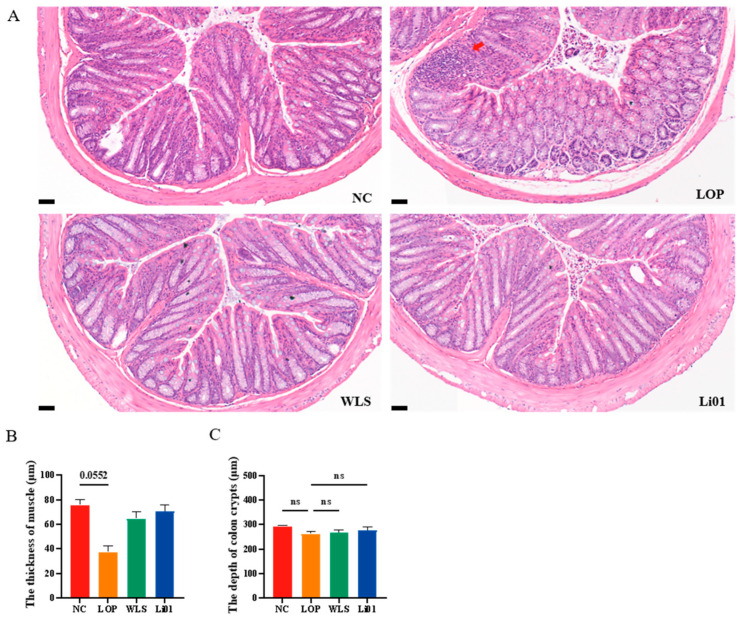
Histologic examination of colon sections in mice with loperamide-induced constipation (LOP) after treatment with *Ligilactobacillus salivarius* Li01 (Li01), Weileshu probiotic (WLS), and the control group (NC). (**A**) The red arrows indicate the inflammatory cells. The scale bars represent 50 µm. (**B**) The thickness of muscle. (**C**) The depth of colon crypts. Data are presented as mean ± SEM, *n* = 3. Significant differences are denoted by “ns” (*p* ≥ 0.05).

Constipation may affect the intestinal tract tissue of mice, resulting in inflammation and incomplete intestinal structure [40]. Histologic examination of colonic tissues is shown in Figure 3. A small number of inflammatory cells were observed infiltrating the colonic tissue of mice in the LOP group, and the colonic muscularis was decreased in thickness compared to mice in the NC group (*p* = 0.0552). No significant variation was found in goblet cells and crypts in the LOP, Li01, and WLS groups compared to the NC group. The colonic tissues of Li01 and WLS mice showed no obvious pathological alterations, and each tissue layer was clearly visible. These results indicate that treatment of Li01 may prevent the damage caused by loperamide and help to maintain the health of the GI tract.

### 3.4. Alteration of the 5-HT Signaling Pathway

**Figure 4:**
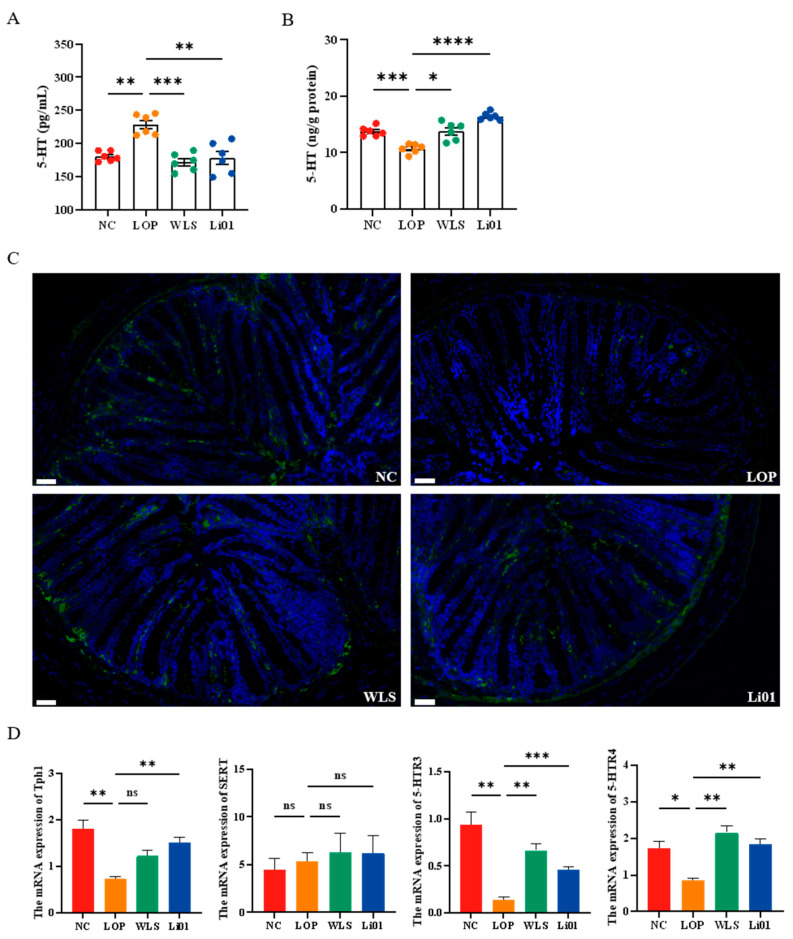
Determination of 5-hydroxytryotamine (5-HT) signaling pathway in mice with loperamide-induced constipation (LOP) after treatment with *Ligilactobacillus salivarius* Li01 (Li01), Weileshu probiotic (WLS), and the control group (NC). The levels of 5-HT in serum (**A**) and colon tissue (**B**) were measured. (**C**) Representative fluorescent images of 5-HT in colonic tissues. The scale bars represent 30 µm. (**D**) The mRNA expression levels of Tph1, SERT, 5-HTR3, and 5-HTR4. Data are presented as mean ± SEM, *n* = 6. Significant differences are denoted by “ns” (*p* ≥ 0.05), * (*p* < 0.05), ** (*p* < 0.01), *** (*p* < 0.001), and **** (*p* < 0.0001).

Several studies have reported elevated 5-HT levels in the colonic tissues of constipated patients [41,42], whereas other studies have found that the deficiency of 5-HT is related to prolonged gastric emptying [43]. To investigate the changes in levels of 5-HT in constipated mice, we quantified 5-HT both in serum and colonic tissues. As shown in Figure 4A, the serum 5-HT level of the LOP mice was remarkably higher than that of the NC mice (*p* < 0.01), indicating that loperamide increased the serum levels of 5-HT. Compared to the mice in the LOP group, significantly decreased serum levels were observed in mice treated with Li01 (*p* < 0.01) and WLS (*p* < 0.001). The 5-HT serum levels in mice treated with Li01 and WLS were similar to those in the NC group. Colonic 5-HT levels, on the other hand, while significantly reduced in mice with loperamide-induced constipated mice compared to mice in the NC group (*p* < 0.001), were higher in mice treated with Li01 (*p* < 0.05) and WLS (*p* < 0.0001) compared to mice in the LOP group (Figure 4B).

5-HT is mainly derived from EC cells in the GI tract, which can act directly on the smooth muscle and promote tissue contraction [43]. Since 5-HT is synthesized by Tph1 in EC cells and migrates into enterocytes or blood via serotonin reuptake transporters (SERTs), fluctuating levels of 5-HT are highly dependent on the levels of Tph1 and SERTs [44]. In this study, significantly lower levels of 5-HT and Tph1 mRNA expression (*p* < 0.01) were observed in colonic tissues of mice in the LOP group compared to mice in the NC group, while the levels of 5-HT and Tph1 mRNA increased in mice treated with the Li01 compared to mice in the LOP group (*p* < 0.01) (Figure 4C,D). Increased level of Tph1 could be one of the main causes leading to the decreased levels of 5-HT in the colon. In mice of the LOP group, more 5-HT enter the bloodstream so that less 5-HT is retained in the colonic tissue. Although it has been reported that the fluctuation of 5-HT in serum is related to SERT by which 5-HT is transported from the gut to the veins [42], we did not observe significant differences in SERT receptors among groups. Therefore, it may be necessary to further study the cause and effect of changes in serum 5-HT.

In order to further explore the effect of altered 5-HT levels on constipation, the expression of 5-HTR3 and 5-HTR4 was investigated. 5-HT can modulate gut secretion, peristalsis as well as vasodilation by activating a variety of 5-HT receptors [45]. 5-HTR3 and 5-HTR4 are important 5-HT excitatory receptors, which are widely present in the GI tract. 5-HTR3 has been confirmed to be relevant to gastrointestinal transmission and for controlling the passage rate of ingested food contents in the GI tract [30]. 5-HTR4 is a G-protein-coupled receptor that can stimulate gut motility and fluid secretion and treat constipation-related symptoms [11,46]. Both 5-HTR3 and 5-HTR4 agonists enhance GI emptying. As shown in Figure 4D, compared to mice in the NC group, mice in the LOP group showed significantly decreased mRNA levels of 5-HTR3 (*p* < 0.05) and 5-HTR4 (*p* < 0.01), while in constipated mice treated with Li01, the levels of 5-HTR3 and 5-HTR4 were higher than in mice in the LOP group (*p* < 0.01, *p* < 0.01). In mice of the WLS group, the levels of 5-HTR3 and 5-HTR4 were also significantly higher than in mice in the LOP group (*p* < 0.001, *p* < 0.01). Therefore, our data demonstrate that Li01 can reverse the reduction of 5-HT through improving de novo synthesis in colonic tissue during constipation and that the 5-HT signaling pathway may be the main mechanism by which Li01 alleviates constipation-related symptoms.

### 3.5. Alteration of the Gut Microbiota Composition

**Figure 5:**
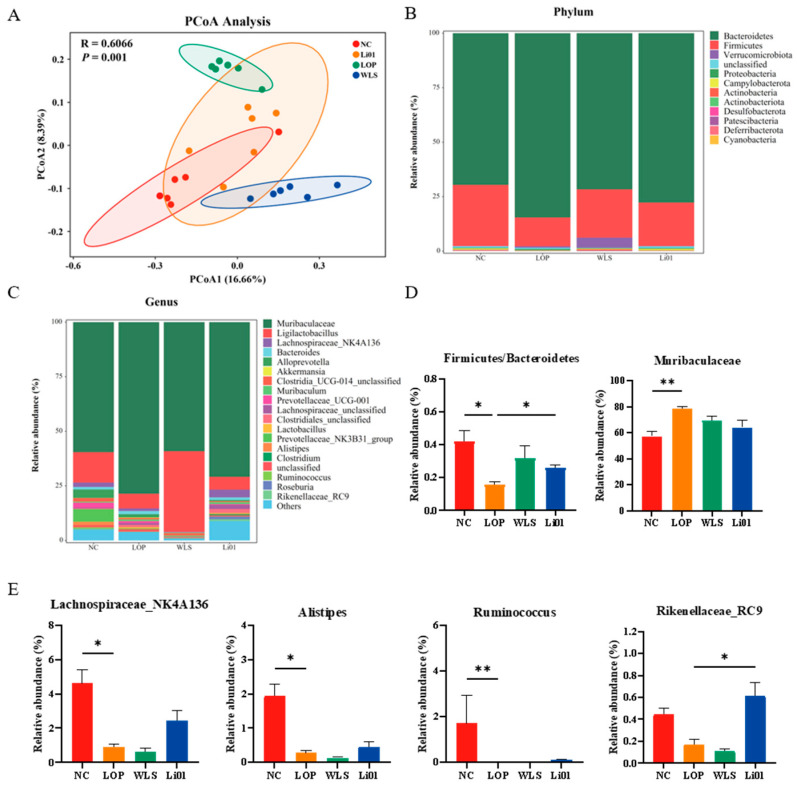
Alteration of the gut microbiota composition of mice in the normal, control group (NC), loperamide-induced constipation group (LOP), Weileshu probiotic group (WLS), and *Ligilactobacillus salivarius* Li01 group (Li01). The relative abundance of the gut microbiota at the phylum level (**A**) and the genus level (**B**) is shown for the different groups. (**C**) β-diversity was evaluated by a principal components analysis (PCoA) of unweighted unifrac distance. (**D**,**E**) The gut microbiota composition of mice in the NC group and the LOP group detected by 16S rRNA gene sequencing. Data are presented as mean ± SEM, *n* = 6. Significant differences are denoted by * (*p* < 0.05), and ** (*p* < 0.01).

An increasing number of studies have demonstrated that intestinal microbiota plays an important role in human health and the development of chronic diseases [47,48]. Alteration of the gut microbiota is also associated with relief or aggravation of constipation symptoms [49] since the gut microbiota can interact with the neuroendocrine system through 5-HT signaling and its metabolites, which affect intestinal motility and intestinal inflammatory response [50,51]. Therefore, in this study, the effects of the probiotic Li01 on the modulation of the gut microbiota of constipated mice were also evaluated (Figure 5).

The β-diversity of the gut microbiota in mice was explored by using principal coordinates analysis (PCoA). As shown in Figure 5A, significant differences in microbial composition could be observed among mice in the NC, LOP, WLS, and Li01 groups (*p* = 0.001). The fecal samples of the healthy mice were dominated in abundance by bacteria from the phyla *Bacteroidetes* and *Firmicutes*, although bacteria from *Verrucomicrobiota*, *Proteobacteria*, and *Campylobacterota* were also present (Figure 5B). *Firmicutes* are generally regarded to be associated with a faster GI transit [52]. In our study, after constipation was induced, the ratio of *Firmicutes/Bacteroidetes* significantly decreased (*p* < 0.01) in mice in the LOP group compared with mice in the NC group. This was reversed after Li01 treatment (*p* < 0.05) (Figure 5D). These results are consistent with previous studies in which constipated mice showed an elevated abundance of *Bacteroidetes* and a decreased abundance of *Firmicutes* [53]. At the genus level (Figure 5C–E), after constipation was induced, when comparing the relative abundance genera in mice in the LOP group with mice in the NC group, an increase in *Muribaculaceae* (*p* < 0.01) and a decrease in *Lachnospiraceae_NK4A136* (*p* < 0.05), *Alistipes* (*p* < 0.05), and *Ruminococcus* (*p* < 0.01) were observed. *Lachnospiraceae_NK4A136* has been reported to have anti-inflammatory properties and contribute to the repair of damaged intestinal mucosa [54]. Qiao et al. found that the abundance of *Ruminococcus* was decreased in constipated mice, which is consistent with our findings [55]. On the contrary, compared to mice in the LOP group, an increased abundance of *Rikenellaceae_RC9* (*p* < 0.05) and a slightly decreased abundance of *Muribaculaceae* (*p* = 0.09) were observed in mice in the Li01 group. Bacteria belonging to *Rikenellaceae_RC9* have been reported to be able to reduce inflammation by stimulating immune regulation [56]. From our results, we inferred that Li01 may alter the composition of the gut microbiota, which contributes to the relief of inflammation induced by constipation.

## 4. Conclusions

**Figure 6:**
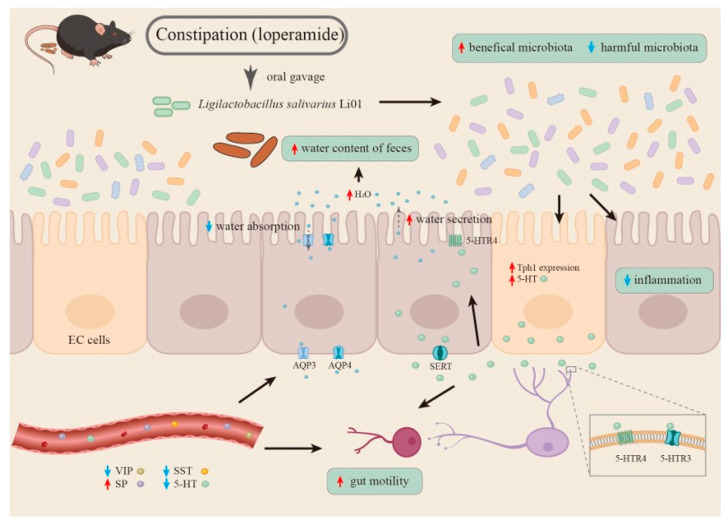
A summary of possible mechanisms by which Li01 can alleviate constipation symptoms.

A summary of possible mechanisms by which Li01 can relieve constipation is presented in Figure 6. In short, our data show that the gut microbiota can be modulated by Li01 intervention in mice with loperamide-induced constipation by increasing the ratio of *Firmicutes*/*Bacteroidetes* and the abundance of genera *Rikenellaceae_RC9.* Li01 changes the 5-HT signaling, with increased expression of Tph1 and 5-HTR3 and 5-HTR4, leading to elevated levels of 5-HT in the colon and enhanced gut motility and fluid secretion. The measured serum parameters in mice with constipation were also altered. Serum levels of VIP and SST were reduced, and SP levels increased, which may down-regulate the AQP3 and AQP4 expressions in the colonic tissue leading to lubrication of the intestinal tract and promoting the GI transit of feces. However, further studies utilizing a combination of metabolomics, immunological techniques, and germ-free mouse models are still needed to clarify the mechanisms by which Li01 can alleviate constipation symptoms.

## Figures and Tables

**Table 1:** Primers used to quantify mRNA in colonic tissue of mice in the experimental model with PCR.

Gene	Forward Primer (5′–3′)	Reverse Primer (5′–3′)
GAPDH	CAGTGGCAAAGTGGAGATTGTTG	TCGCTCCTGGAAGATGGTGAT
AQP3	GCCAAGGTAGGATAGCAAATAA	TTGAAAACTTGGTCCCTTGC
AQP4	CACCATAAACTGGGGTGGCT	AGACGGACTTAGCGATGCTG
Tph1	CCATCTTCCGAGAGCTAAACAAA	TCTTCCCGATAGCCACAGTATT
SERT	GCGACGTGAAGGAAATGCTG	GGAGTTGGGGTGGACTCATC
5-HTR3	CTGTGGCGATCACCGGAAG	GGCTGACTGCGTAGAATAAAGG
5-HTR4	TCGATCTTTCACCTGTGCTGTAT	TGTTGTTCCAGCCTTGCATTATG
Muc2	AAGCTGCACGGACACCTCTATAT	TCGCTCTTGGTCAGGACATAGTA

AQP3: aquaporin 3; AQP4: aquaporin 4; Tph1: tryptophan hydroxylase 1; SERT: serotonin reuptake transporter; 5-HTR3: 5-hydroxytryptamine receptor 3A; 5-HTR4: 5-hydroxytryptamine receptor 4; Muc2: mucin 2.

## Data Availability

Not applicable.
